# *LMOf2365_0442* Encoding for a Fructose Specific PTS Permease IIA May Be Required for Virulence in *L. monocytogenes* Strain F2365

**DOI:** 10.3389/fmicb.2017.01611

**Published:** 2017-08-29

**Authors:** Yanhong Liu, Brian B. Yoo, Cheng-An Hwang, Yujuan Suo, Shiowshuh Sheen, Parvaneh Khosravi, Lihan Huang

**Affiliations:** ^1^Molecular Characterization of Foodborne Pathogens Research Unit, Eastern Regional Research Center, Agricultural Research Service, United States Department of Agriculture, Wyndmoor PA, United States; ^2^Clinical and Environmental Microbiology Branch, Division of Healthcare Quality and Promotion, National Center for Emerging and Zoonotic Infectious Diseases, Centers for Disease Control and Prevention, Atlanta GA, United States; ^3^Residue Chemistry and Predictive Microbiology Research Unit, Eastern Regional Research Center, Agricultural Research Service, United States Department of Agriculture, Wyndmoor PA, United States; ^4^Institute for Agri-Food Standards and Testing Technology, Shanghai Academy of Agricultural Sciences Shanghai, China; ^5^Food Safety Intervention Technologies Research Unit, Eastern Regional Research Center, Agricultural Research Service, United States Department of Agriculture, Wyndmoor PA, United States

**Keywords:** *L. monocytogenes* invasion assays, plaque forming assays, phosphotransferase transport system (PTS), virulence gene expression, RT-PCR

## Abstract

*Listeria monocytogenes* is a foodborne pathogen that causes listeriosis, which is a major public health concern due to the high fatality rate. *LMOf2365_0442, 0443*, and *0444* encode for fructose-specific EIIABC components of phosphotransferase transport system (PTS) permease that is responsible for sugar transport. In previous studies, in-frame deletion mutants of a putative fructose-specific PTS permease (*LMOf2365_0442, 0443*, and *0444*) were constructed and analyzed. However, the virulence potential of these deletion mutants has not been studied. In this study, two *in vitro* methods were used to analyze the virulence potential of these *L. monocytogenes* deletion mutants. First, invasion assays were used to measure the invasion efficiencies to host cells using the human HT-29 cell line. Second, plaque forming assays were used to measure cell-to-cell spread in host cells. Our results showed that the deletion mutant Δ*LMOf2365_0442* had reduced invasion and cell-to-cell spread efficiencies in human cell line compared to the parental strain *LMOf2365*, indicating that *LMOf2365_0442* encoding for a fructose specific PTS permease IIA may be required for virulence in *L. monocytogenes* strain F2365. In addition, the gene expression levels of 15 virulence and stress-related genes were analyzed in the stationary phase cells of the deletion mutants using RT-PCR assays. Virulence-related gene expression levels were elevated in the deletion mutants Δ*LMOf2365_0442-0444* compared to the wild type parental strain *LMOf2365*, indicating the down-regulation of virulence genes by this PTS permease in *L. monocytogenes*. Finally, stress-related gene *clpC* expression levels were also increased in all of the deletion mutants, suggesting the involvement of this PTS permease in stress response. Furthermore, these deletion mutants displayed the same pressure tolerance and the same capacity for biofilm formation compared to the wild-type parental strain *LMOf2365*. In summary, our findings suggest that the *LMOf2365_0442* gene can be used as a potential target to develop inhibitors for new therapeutic and pathogen control strategies for public health.

## Introduction

*Listeria monocytogenes* is a Gram-positive intracellular human pathogen that can cause listeriosis with a high mortality rate (20–30% in immuno-compromised groups). It is widely distributed in soil and food environments. *L. monocytogenes* is also a foodborne pathogen that is often associated with a variety of raw and processed food products, including milk, meat, and vegetables. It can persist in food processing environments for years since it can form biofilms and survive under harsh conditions such as low pH and low temperature (Farber and Peterkin, 1991).

The virulence of *L. monocytogenes* involves host cell adhesion and invasion, escape from vacuoles, intracellular multiplication, and cell-to-cell spreading (Camejo et al., 2011). A number of genes associated with virulence have been identified in *L. monocytogenes*. For example, *prfA* encodes a transcriptional regulator that activates the transcription of a number of virulence genes such as *hly, plcA, plcB*, and *inlA* in *L. monocytogenes*. *SigB* also encodes a transcriptional regulator that positively regulates the transcription of stress-related genes such as *clpC* and *clpE* (Hu et al., 2007). A number of genes involved in adhesion include *actA, ami, fbpA*, and *flaA* (Dons et al., 2004; Camejo et al., 2011). Internalin A and B (*inlA* and *inlB*) mediate invasion into mammalian cells (Carvalho et al., 2014). *hly* encodes for listeriolysin O, which is responsible for the escape of *L. monocytogenes* from vacuoles. *plcA* and *plcB* are also involved in escape from vacuoles. The *actA* and *iap* genes are involved in intracellular motility and cell-to-cell spread (Camejo et al., 2011).

The phosphotransferase transport system (PTS) serves as a sugar transport system in bacteria. There are approximately 30 copies of different PTS systems present in the genome of *L. monocytogenes* (Stoll and Goebel, 2010). A typical PTS contains enzyme I (EI), II (EII), and a heat-stable protein (HPr). EI and HPr are common, whereas EII is sugar-specific and is usually comprised of three domains (IIA, IIB, and IIC). EIIA and EIIB proteins are soluble and hydrophilic whereas EIIC is hydrophobic membrane protein. EIIA interacts with EIIB and EIIB interacts with EIIC (Deutscher et al., 2006). PTSs not only function as carbohydrate transporters, but also regulate numerous cellular processes, including carbon catabolite repression (CCR) (Deutscher et al., 2006). Some PTSs have been associated with stress response and biofilm formation (Wu et al., 2012). *LMOf2365_0442* (encoding for the PTS system, fructose-specific, IIA component), *LMOf2365_0443* (encoding for the PTS system, fructose-specific, IIB component), and *LMOf2365_0444* (encoding for the PTS system, fructose-specific, IIC component) were highly induced under high hydrostatic pressure treatment (Liu et al., 2011) and inhibited under salt stress (Bae et al., 2012) as tested by microarray and real-time PCR assays. In our previous study, mutants with in-frame deletions of these genes had different growth patterns under multiple stress conditions, including acid and salt stresses, indicating that these genes may contribute to the general stress response (Liu et al., 2013). Since a mannose PTS permease has been shown to be related to virulence gene expression in *L. monocytogenes* (Vu-Khac and Miller, 2009), we hypothesized that the *LMOf2365_0442, 0443*, and *0444* encoding for PTS permease might also be involved in virulence in *L. monocytogenes*.

In this study, the three in-frame *L. monocytogenes* deletion mutants (Δ*LMOf2365_0442-0444)* were tested for their virulence potential using invasion and plaque forming assays. The virulence and stress-related gene expression levels of deletion mutants were also examined in stationary-phased cells using RT-PCR assays. Finally, the pressure tolerance and the biofilm formation ability of these deletion mutants were also determined.

## Materials and Methods

### Bacterial Strains and Human Cell Line (HT-29) Culture Conditions

*Listeria monocytogenes* strain F2365 (*LMOf2365*) isolated from Mexican-style soft cheese was implicated in an outbreak of listeriosis in California in 1985 (Linnan et al., 1988). It was used in this study since its genome is fully sequenced and annotated (Nelson et al., 2004). Glycerol stock cultures of *L. monocytogenes* F2365 (serotype 4b), *L. innocua* (ATCC^®^ 51742^TM^), and three isogenic deletion mutants Δ*LMOf2365_0442-0444* of the parent strain *L. monocytogenes* F2365 (Liu et al., 2013) stored at -80°C were streaked onto Brain Heart Infusion (BHI) (Sigma–Aldrich St. Louis, MO, United States) agar plates. Single colonies picked from agar plates were grown to logarithmic or stationary phases prior to each experiment.

The human adenocarcinoma cell line HT-29 purchased from ATCC (Manassas, VA, United States) was grown in 75-cm^2^ plastic tissue culture flasks (Falcon, Durham, NC, United States) in Dulbecco’s Modified Eagle’s Medium (DMEM) with glucose (4.5 g/L) (Invitrogen, Carlsbad, CA, United States) supplemented with 10% (v/v) fetal bovine serum (Invitrogen) and 1 mM sodium pyruvate (Invitrogen). Antibiotics (100 IU/ml penicillin and 100 μg/ml streptomycin) were routinely added to the culture medium except for the medium used 24 h prior to the invasion and plaque forming assays. Cells were maintained in a humidified incubator at 37°C under 5% (v/v) CO_2_.

### Invasion Assays

Invasion assays were performed to assess the virulence of *Listeria* strains according to Roche et al. (2005) with the following modifications. HT-29 human cells (ATCC HTB-38) were grown on 24-well tissue culture plates for 5 days to obtain almost confluent monolayers. Strains of *L. monocytogenes* (isogenic deletion mutants Δ*LMOf2365_0442*,Δ*LMOf2365_0443*, Δ*LMOf2365_0444* and the parental *LMOf2365*) and *L. innocua* grown to log-phase at 37°C were used for invasion assays. HT-29 cell monolayers incubated in DMEM medium without antibiotics for 24 h were infected for 1 h at 37°C with 10^7^ bacterial cells in 300 μl BHI liquid medium [Multiplicity of Infection (MOI) = 60]. The cell monolayers were washed with DMEM and incubated in DMEM containing gentamicin (100 μg/ml) for 1.5 h at 37°C. The cell monolayers were gently washed three times with phosphate buffered saline (pH 7.4) and then disrupted with 1 ml cold sterile water (4°C). Viable intracellular bacteria were counted after plating serial dilutions on BHI agar plates. The results were expressed as log numbers of CFU recovered relative to the number of bacteria (10^7^) deposited per well. Each experiment was conducted in duplicate and repeated three times for each strain.

### Plaque-Forming Assays

Plaque forming assays were performed using HT-29 cells according to Roche et al. (2001). Briefly, HT-29 cells were grown on 24-well tissue culture plates for 5 days to obtain almost confluent monolayers. HT-29 cell monolayers were incubated in medium without antibiotics for 24 h. Strains of *L. monocytogenes* (isogenic deletion mutants Δ*LMOf2365_0442*,Δ*LMOf2365_0443*,Δ*LMOf2365_0444* and parental *LMOf2365*) grown to log-phase at 37°C were used to infect HT-29 cell monolayers with a dilution series of 10^2^ to 10^7^ cells per well, and they were incubated for 2 h at 37°C. *L. innocua* was used as a negative control. After removal of the bacterial suspensions, cell monolayers were washed with DMEM and incubated in DMEM containing 100 μg/ml of gentamicin for 1.5 h. Each well was covered with 400 μl DMEM with 10 μg/ml gentamicin containing 0.5% agarose. After solidification, the same liquid medium (400 μl) was added to the top of the agar to prevent starvation. Tissue culture plates were incubated overnight at 37°C under 5% (v/v) CO_2_. The cells were stained with 0.01% neutral red solution in DMEM medium with 0.5% agarose and were incubated at 37°C for overnight. Enumeration of formed plaques was performed using an inverted microscope. The results were expressed as log numbers of plaques per 10^7^ bacteria deposited per well. Experiments were carried out in duplicate and repeated three times for each strain.

### RT-PCR Analysis of Virulence and Stress-Related Genes

The deletion mutant (Δ*LMOf2365_0442*,Δ*LMOf2365_0443*, Δ*LMOf2365_0444*) strains together with parental strain *LMOf2365* were grown to stationary phase at 37°C. Total RNA was isolated from stationary phase (normal growth conditions) for bacterial cells of the deletion mutant strains, as well as the wild-type *L. monocytogenes* F2365 parent strain (**Table 1**) as previously described (Liu and Ream, 2008). Primers targeting the genes related to virulence and stress response (**Table 1**) were designed using Primer3 (v.0.4.0) software^1^ based on the gene sequences of *L. monocytogenes* strain F2365 (GenBank accession#AE017262). The specificity of the primer sequences was further determined using the NCBI BLASTN program against the non-redundant (nr) database, and analyses revealed that the primer sequences showed 100% homology only to *L. monocytogenes* strain F2365 (GenBank accession#AE017262). Primers were also designed to the *spoG* housekeeping gene used as an internal control (**Table 1**). cDNA synthesis and real-time PCR analysis were performed as described previously (Liu and Ream, 2008). Reactions without reverse transcriptase were used as negative controls.

**Table 1:** Oligonucleotides used for real-time PCR to evaluate the virulence and stress-related genes in *Listeria monocytogenes*.

GENE	Forward primer sequence	Reverse primer sequence	Amplicon size (bp)
*actA*	AAGAGTTGAACGGAGAGGT	TCAGCTAGGCGATCAATTTC	121
*ami*	GTAACCATTCGCGATGACTC	CTTGAATAGCGAACCCTTGA	100
*clpC*	GTAACCATTCGCGATGACTC	CTTGAATAGCGAACCCTTGA	100
*clpE*	CAGAAGCACTAACAGCAGCA	TCACCGTATTTTCGTCCAGT	141
*fbpA*	GCGGTCGAAGTAGTGAAAGA	AGCTAGTTCTTGGCGGATTT	126
*flaA*	CGCAAGAACGTTTAGCATCT	ATGGATGAGTTTTTGCTTGC	127
*hly*	GAATGCAATTTCGAGCCTAA	AGTCATTCCTGGCAAATCAA	133
*iap*	GAAAAACAAGCTGCACCAGT	CTGTTGGTGCTTTAGGTGCT	109
*inlA*	ATGGGATTTTGCGACAGATA	CGGAAGGTGGTGTAGTGTTC	143
*inlB*	ACCTAAACCTCCGACCAAAC	TCGTTTCCGCTTTAAACATC	140
*lap*	ATCCCTTCCCTAACACTTGG	GTGGAAGTTTGAACCATTGC	133
*plcA*	AAGACGAGCAAAACAGCAAC	CTCGTGTCAGTTCTGGGAGT	100
*plcB*	ATCCTATCCACCAGGCTACC	TCTTTCACGTCATTTGAGCA	117
*pfrA*	CGCAAGAACGTTTAGCATCT	ATGGATGAGTTTTTGCTTGC	127
*sigB*	TCGCAAATATTCCCAAGGTA	TGACGGTGAATTCCGTGATA	127
*spoG*	TGACGGTGAATTCCGTGATA	TCAGCAGAAACGGATTCAGA	147

### High Pressure Processing (HPP) Treatments

The deletion mutant Δ*LMOf2365_0442*, 0443, 0444 strains, together with wild-type *L. monocytogenes* F2365 parent strain were grown to log phase (with optimal densities of OD_600__nm_ between 0.3 and 0.5) at 30°C. Prior to the high pressure treatments, 2 ml of bacterial cultures were taken for plate counts. Twenty milliliters of bacterial cultures were vacuum-sealed in two plastic bags and subject to high pressure treatments (400 and 450 MPa, respectively) at 4°C for 3 min. HPP was performed in a laboratory scale pressure unit (Mini Food lab FPG5620, Stansted Fluid Power Ltd., Essex, United Kingdom), comprised of a double-jacketed thick-wall stainless steel cylinder (approximate volume of 0.3 L) having an internal stainless steel sample holder of 25.4 mm × 254 mm (diameter × length). The thick-wall cylinder was maintained at a set-point temperature in which heat transfer fluid continuously circulated from a refrigerated liquid chiller (Proline RP 855, Lauda, Germany). The refrigerated chiller was set at 4°C, which indirectly cooled the pressure transmitting medium (a mixture of ethanol and castor oil, 80%/20% weight basis). The pressure come-up rate was 100 MPa per 15 s (or 6.67 MPa/s), and the release rate was 100 MPa per 9 s (or 11.11 MPa/s) (Hsu et al., 2015). This temperature set-up ensured that foods in the pressure chamber were maintained at <40°C during the HPP treatments and eliminated the potential for thermal lethality. After pressure treatments, 2 ml of the suspension was used for plate counting.

### Biofilm Formation Assays

Biofilm assays were performed as described (Liu et al., 2017) with the following modifications. Single colonies of *L. monocytogenes* F2365, Δ*LMOf2365_0442*-0444, and *L. innocua* were inoculated into 5 ml of BHI broth and incubated at 37°C overnight with agitation at 200 rpm. The bacterial overnight cultures were diluted 100-fold in Modified Welshimer’s Broth (MWB) (HiMedia Laboratories, Mumbai, India) with glucose as the sole carbon source. Two hundred microliters of diluted bacterial cultures were added to a 96-well PVC microtiter plate previously rinsed with 70% ethanol. For each strain, 200 μL of the freshly diluted culture were placed in eight different wells. Two hundred microliters of MWB (eight wells) was used as a negative control. The 96-well PVC microtiter plate was incubated at 30°C in a humidified container for 48 h. After removal of the medium, the plate was washed five times with distilled water and air dried for 45 min. The plate was stained with 200 μL of 0.1% crystal violet for 45 min and washed five times with distilled water. After 30 min of destaining with 200 μL of 95% ethanol for 30 min, the absorbance at OD_595__nm_ was measured using a Tecan Safire 2 microplate reader (Tecan Group Ltd., Switzerland). The absorbance readings at OD_595__nm_ were normalized by subtracting the medium only OD_595__nm_ numbers. Any absorbance at OD_595__nm_ above 0 indicated some biofilm formation. Three independent experiments were performed.

### Statistical Analysis

Data collected from the study were analyzed using the Student’s *t*-test of the Statistical Analysis Software (SAS Institute Inc., Cary, NC, United States) for paired comparison with *P* < 0.05 considered significant.

## Results

### Deletion Mutant Δ*LMOf2365_0442* Displayed Reduced Invasion and Plaque Forming Efficiencies in HT-29 Cell Line

**FIGURE 1:**
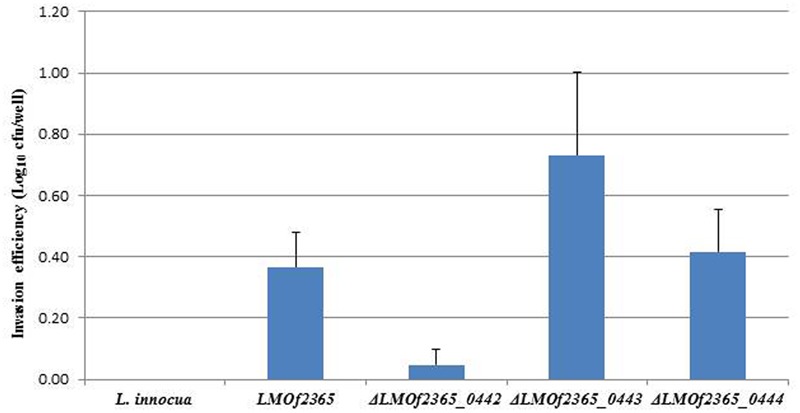
Invasion of *Listeria monocytogenes* and *L. innocua* strains in HT-29 cells. HT-29 cell monolayers were incubated with *Listeria* strains grown to log-phase for invasion assays. Viable intracellular bacteria were counted after plating serial dilutions on BHI agar plates. The results were expressed as log numbers of CFU recovered relative to the number of bacteria (10^7^) deposited per well. Mean values and standard deviations were calculated from three independent experiments.

**FIGURE 2:**
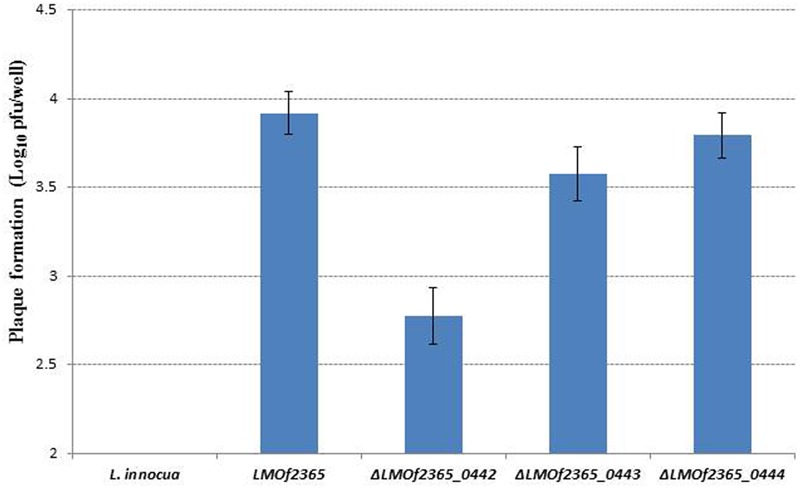
Plaque formation of *Listeria* strains in HT-29 monolayers. Results are presented as the log_10_ numbers of plaque-forming units (pfu) per well ± standard deviation of triplicate.

To understand if the PTS EII complex *LMOf2365_0442-0444* was involved in host infection, two *in vitro* assays (cell invasion and plaque forming assays) using HT-29 cell monolayers were used to test the virulence potential for each deletion mutant. As shown in **Figure 1**, *L. monocytogenes* F2365 (*LMOf2365*), which was used as a positive control, had high invasion efficiency (0.36 log_10_ cfu/well). The deletion mutant strain Δ*LMOf2365_0442* showed deficiency in invasion (14%) whereas Δ*LMOf2365_0443 and*Δ*LMOf2365_0444* had higher invasion efficiencies (203 and 117%, respectively) compared to the wild-type strain *LMOf2365* (100%). Non-pathogenic strain *L. innocua* that served as a negative control had no invasion. The second *in vitro* assay for virulence was based on the ability of *Listeria* strains to form plaques on HT-29 cell monolayers. As shown in **Figure 2**, *L. monocytogenes* F2365 formed a higher number of plaques (approximately 3.9 log_10_ pfu/well) compared to non-pathogenic strain *L. innocua*, which did not form any plaques. Δ*LMOf2365_0442* formed a lower number of plaques (2.8 log_10_ pfu/well) whereas Δ*LMOf2365_0443* and Δ*LMOf2365_0444* had similar plaque forming abilities (3.6 and 3.8 log_10_ pfu/well, respectively) as the wild type *LMOf2365* (3.9 log_10_ pfu/well). Taken together, results from invasion and plaque forming assays demonstrated that deletion mutant Δ*LMOf2365_0442* showed a deficiency in both invasion and intracellular cell-to-cell spread in HT-29 cell monolayers, suggesting that *LMOf2365_0442* is required for virulence in *L. monocytogenes*.

### Virulence and Stress-Related Gene Expression Levels Were Elevated in the Deletion Mutants

Fifteen genes related to virulence and stress response (Olesen et al., 2009) were chosen for real-time PCR assays to determine the gene expression levels in deletion mutants under stationary-phase conditions. Compared to the wild-type parental *LMOf2365* strain, the majority of the virulence genes were up-regulated in the deletion mutants (**Table 2**), indicating that the PTS permease (*LMOf2365_0442*-*0444*) negatively regulated virulence gene expression. Of the virulence genes, the expression level of *pfrA*, the major virulence regulator in *L. monocytogenes*, was moderately high (3.4 to 4.6-folds) compared to the wild-type parental strain *LMOf2365*. Genes (*actA, plcA, plcB*, and *hly*) regulated by *pfrA* were also elevated in Δ*LMOf2365_0442* and Δ*LMOf2365_0444.* Interestingly, the gene expression levels of *hly, lap, actA*, and *plcB* had little change in Δ*LMOf2365_0443.* Our previous studies indicated that the deletion mutants were more resistant to multiple stress conditions (Liu et al., 2013), indicating that they may contribute to a general stress response. RT-PCR assays were used to determine the gene expression levels of three stress-related genes (*sigB, clpC*, and *clpE*). As shown in **Table 2**, the expression levels of *clpC* were elevated in all of the three deletion mutants. The expression levels of *clpE* and *sigB* were moderately elevated (3.5- and 4.8-folds) in Δ*LMOf2365_0444 and*Δ*LMOf2365_0442*, respectively. The increased levels of stress-related gene expression confirmed our previous observation that these deletion mutants may contribute to general stress response (Liu et al., 2013).

**Table 2:** Relative changes in expression levels for virulence and stress–related genes in *L. monocytogenes* deletion mutants.

	*actA*	*ami*	*iap*	*inlA*	*inlB*	*lap*	*fbaA*	*plcA*	*plcB*	*prfA*	*hly*	*flaA*	*clpC*	*clpE*	*sigB*
*LMOf2365*	1	1	1	1	1	1	1	1	1	1	1	1	1	1	1
Δ*LMOf2365_0442*	3.2^a^	10.6	10.2	22.6	13.9	26.9	5.5	5.3	4.1	3.4	3.7	5.3	4.6	1.3	4.8
Δ*LMOf2365_0443*	-1.1	10.9	2.2	6.7	8.9	-1.1	4.0	2.2	1.1	10.6	-1.6	21.1	4.1	1.5	1.4
Δ*LMOf2365_0444*	4.6	8.6	5.7	15.5	19.0	4.8	4.9	5.5	4.3	2.0	5.3	3.9	3.4	3.5	1.6

*^a^Numbers represent the transcript ratios between deletion mutants and *LMOf2365* as determined by real-time PCR. Data shown are average values of three replicates.*

### Deletion Mutant Δ*LMOf2365_0442-0444* Strains Displayed the Same Tolerance to High Pressure Compared to the Parental *LMOf2365* Strain

**FIGURE 3:**
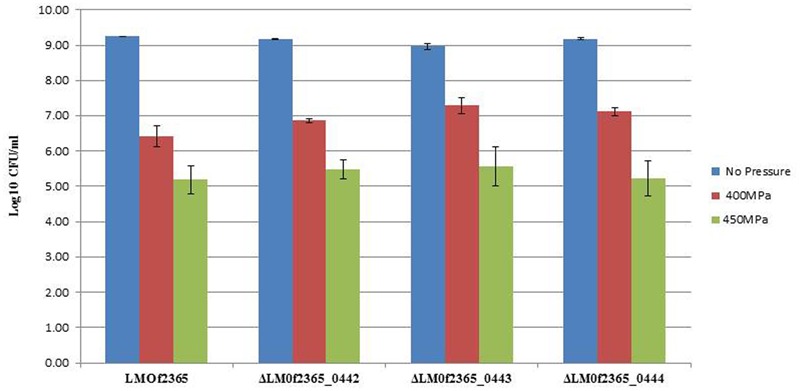
Growth of *L. monocytogenes* strains before and after high pressure treatments. The log phased bacterial cells were subjected to high pressure treatments (400 and 450 MPa, respectively) at 4°C for 3 min. The results were expressed as log numbers of cfu/ml ± standard deviations of three independent experiments.

Since *LMOf2365_0442-0444* were highly induced under high pressure (Liu et al., 2011), we would like to know whether deletion of these genes will change their pressure tolerance. To test whether the PTS operon had an effect on high pressure tolerance, the deletion mutants, together with parental *LMOf*2365 strains, were subject to high pressure treatments (450 and 400 MPa for 3 min). As shown in **Figure 3**, the wild-type *L. monocytogenes* F2365 showed ∼3 and 4 log reduction under 400 and 450 MPa, respectively. The deletion mutants showed similar log reductions compared to the wild-type strain.

### Depletion of PTS Permease Had No Effect on Biofilm Formation

**FIGURE 4:**
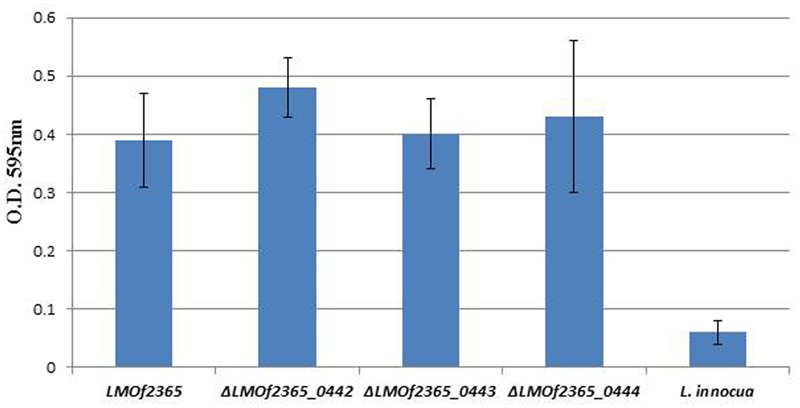
Biofilm formation in *Listeria* strains. *Listeria* strains were tested for biofilm formation in Modified Welshimer’s Broth (MWB). The biofilm was assessed by crystal violet staining and quantified at OD_595__nm_ using a spectrophotometer. The results were expressed as the mean absorbance at OD_595__nm_ ± standard deviations of eight replicates.

A fructose PTS required for virulence in *Streptococcus gordonii* was involved in biofilm formation (Loo et al., 2003). To determine whether the PTS permease (*LMOf2365_0442-0444)* is involved in biofilm formation, the deletion mutants (Δ*LMOf2365_0442-0444*) were subject to a biofilm assay. As shown in **Figure 4**, the wild-type parental LMOf2365 strain formed biofilm (OD_595__nm_ ∼0.4) under experimental condition, whereas *L. innocua* used as a negative control hardly formed any biofilm (OD_595__nm_ < 0.1). The deletion mutant Δ*LMOf2365_0443* had a similar ability for biofilm formation compared to the wild-type LMOf2365 strain. Although Δ*LMOf2365_0442 and*Δ*LMOf2365_0444* had a slightly increased ability for biofilm formation, the differences were not statistically significant.

## Discussion

In the current study, two *in vitro* assays were used to test the virulence of *L. monocytogenes* deletion mutants. Δ*LMOf2365-0442* was defective in both assays, indicating that this gene is required for virulence. Although we did not test the virulence using the *in vivo* mouse model, previous studies showed that the results from plaque forming and invasion assays in *L. monocytogenes* correlated well with bacterial virulence *in vivo* (Van Langendonck et al., 1998; Roche et al., 2001). Some PTSs have been shown to be involved in virulence in *L. monocytogenes*. For example, a PTS mutant with a deficiency in infection was identified in *L. monocytogenes* through transposon-tagged mutagenesis (Cummins et al., 2013). Using microarrays, a transcriptional profiling of *L. monocytogenes* following epithelial cell infection identified several up-regulated PTS genes (Joseph et al., 2006). We showed for the first time that *LMOf2365_0442* encoding a fructose-specific, permease EIIA component of the PTS is required for virulence in *L. monocytogenes*. Since fructose has been linked to virulence in *Spiroplasma citri* (Gaurivaud et al., 2000), we speculated that *LMOf2365_0442* regulates virulence probably through limiting the fructose intake. Further experiments are needed to test this hypothesis.

In our study, fifteen genes related to virulence and stress previously used to study acid and salt stress (Olesen et al., 2009) were chosen to determine the gene expression levels in deletion mutants. The deletion mutants showed high levels of virulence genes expression compared to the wild type. Our results are consistent with previous study in which it was found that the mRNA levels of *prfA* gene clusters were elevated in a PTS deletion mutant (Aké et al., 2011). Since glucose represents the only carbon source in BHI growth medium, the utilization of glucose is favored through the inhibition of the synthesis of the enzymes involved in the catabolism of other carbon sources. This phenomenon is called CCR (Deutscher, 2008; Görke and Stülke, 2008). Glucose represses virulence gene expression in *L. monocytogenes* (Milenbachs et al., 1997). A mannose-specific PTS that down-regulated *prfA* gene expression was also involved in CCR (Vu-Khac and Miller, 2009). The activity of PrfA seems to be controlled by the phosphorylation state of PTS components (Herro et al., 2005; Poncet et al., 2009). Fructose also repressed virulence genes such as *hly* and *plcA* (Deutscher et al., 2006). It is likely that under normal conditions, the virulence gene expression levels are repressed due to CCR. We propose that the fructose-specific PTS permease (*LMOf2365_0442-0444*) might be used to repress virulence gene expression in *L. monocytogenes*, and deletion of PTS could cause inefficient fructose intake, therefore, deactivating CCR. As a result of CCR deactivation, the virulence gene expression is de-repressed.

In this study, the log-phased bacterial cells were chosen to perform invasion and plaque forming assays since they represented the most virulent stage in *L. monocytogenes* (Dramsi et al., 1993). The deletion mutants showed reduced virulence in terms of invasion and cell-to-cell spreading ability in human cell line HT-29; however, a number of virulence genes showed increase expression under normal growth conditions. This seems contradicting, but the gene expression experiments were performed under normal growth condition, not under infection conditions. It is likely that the virulence gene expression levels were repressed due to catabolite repression under normal growth conditions, however, these genes were de-repressed in Δ*LMOf2365_0442-0444*.

*L. monocytogenes* is a major concern in the food industry. This foodborne pathogen is widely distributed in food products and food processing facilities. In addition, it also forms biofilms in food processing environments, increasing the probability of food contamination and making it even more difficult to eliminate in food. HPP has been utilized in the food industry to control *L. monocytogenes* and extend product shelf life (Gálvez et al., 2007). In our previous study, microarray technology was used to monitor the gene expression profiles of a pressure tolerant mutant under HPP. The gene expression levels of *LMOf2365_0442, 0443*, and *0444* were elevated under high pressure treatment (Liu et al., 2011). Despite the high induction levels of *LMOf2365_0442, 0443*, and *0444* under HPP, the deletion mutants of Δ*LMOf2365_0442, 0443*, and *0444* displayed the same pressure tolerance compared to the parental wild-type *LMOf2365* strain (**Figure 3**). The biofilm forming abilities of these deletion mutants were also tested, our results showed that deletion of *LMOf2365_0442, 0443*, and *0444* genes did not alter biofilm formation in *L. monocytogenes* (**Figure 4**). Although our data suggest that *LMOf2365_0442, 0443* encoding for the EIIABC components of PTS are not involved in biofilm formation in *L. monocytogenes*, the EIIA^Glc^ component of PTS has been shown to play a role in biofilm formation in *Vibrio* cholera (Pickering et al., 2014).

The fact that PTSs are uniquely present only in prokaryotic bacteria but not in eukaryotes may allow PTSs to be a useful drug target. Targeting bacterial virulence is an alternative approach to kill pathogens in the host. We have shown that deletion mutant of *LMOf2365_0442* (encoding for the PTS system, fructose-specific, IIA component) had reduced virulence, inhibitors to the PTS system, fructose-specific, IIA component would attenuate the virulence in *L. monocytogenes*. In addition, PTSs are highly conserved among the prokaryotic bacteria; therefore it is easier to develop inhibitors for PTSs. In fact, inhibitors were developed through *in silico* and library screening approaches (Stephenson and Hoch, 2002).

## Author Contributions

YL designed the experiments and wrote the manuscript. BY, C-AH, YS, SS, PK, and LH did the experiments. All of the authors have made a substantial, direct, and intellectual contribution to the work and approved it for publication.

## Conflict of Interest Statement

The authors declare that the research was conducted in the absence of any commercial or financial relationships that could be construed as a potential conflict of interest.

## Footnotes

1 http://frodo.wi.mit.edu/
